# Neuropsychiatric Ramifications of COVID-19: Short-Chain Fatty Acid Deficiency and Disturbance of Microbiota-Gut-Brain Axis Signaling

**DOI:** 10.1155/2021/7880448

**Published:** 2021-10-05

**Authors:** Elizabeth M. Sajdel-Sulkowska

**Affiliations:** Department of Psychiatry Harvard Medical School, BWH, Boston, MA 02114, USA

## Abstract

COVID-19-associated neuropsychiatric complications are soaring. There is an urgent need to understand the link between COVID-19 and neuropsychiatric disorders. To that end, this article addresses the premise that SARS-CoV-2 infection results in gut dysbiosis and an altered microbiota-gut-brain (MGB) axis that in turn contributes to the neuropsychiatric ramifications of COVID-19. Altered MGB axis activity has been implicated independently as a risk of neuropsychiatric disorders. A review of the changes in gut microbiota composition in individual psychiatric and neurological disorders and gut microbiota in COVID-19 patients revealed a shared “microbial signature” characterized by a lower microbial diversity and richness and a decrease in health-promoting anti-inflammatory commensal bacteria accompanied by an increase in opportunistic proinflammatory pathogens. Notably, there was a decrease in short-chain fatty acid (SCFA) producing bacteria. SCFAs are key bioactive microbial metabolites with anti-inflammatory functions and have been recognized as a critical signaling pathway in the MGB axis. SCFA deficiency is associated with brain inflammation, considered a cardinal feature of neuropsychiatric disorders. The link between SARS-CoV-2 infection, gut dysbiosis, and altered MGB axis is further supported by COVID-19-associated gastrointestinal symptoms, a high number of SARS-CoV-2 receptors, angiotensin-cleaving enzyme-2 (ACE-2) in the gut, and viral presence in the fecal matter. The binding of SARS-CoV-2 to the receptor results in ACE-2 deficiency that leads to decreased transport of vital dietary components, gut dysbiosis, proinflammatory gut status, increased permeability of the gut-blood barrier (GBB), and systemic inflammation. More clinical research is needed to substantiate further the linkages described above and evaluate the potential significance of gut microbiota as a diagnostic tool. Meanwhile, it is prudent to propose changes in dietary recommendations in favor of a high fiber diet or supplementation with SCFAs or probiotics to prevent or alleviate the neuropsychiatric ramifications of COVID-19.

## 1. Introduction

The first case of COVID-19 can be traced to November 17, 2019; on December 8, 2020, the first public COVID-19 vaccination occurred in the UK, followed on December 14, 2020, by vaccination in the USA heralding human achievement in the battle against infectious diseases. However, while vaccines against SARS-CoV-2 combat the spread of the infection, short-term neuropsychiatric ramifications of COVID-19 and post-COVID conditions are soaring [1]. According to the World Health Organization, more than 30% of severe COVID-19 cases are associated with neuropsychiatric symptoms [2], and they are not limited to patients who had severe COVID-19 [3]. Some reports suggest that more than 80% of hospitalized patients may have neurologic symptoms such as myalgia, headache, encephalopathy, and dizziness; dysgeusia and anosmia are less common [4]. Presently, the underlying mechanisms involved in the long-term neuropsychiatric complications of COVID-19 are not clear.

Neuropsychiatric disorders associated with COVID-19 have been independently linked to changes in the gut microbiota, altered microbiota-gut-brain (MGB) axis, and brain inflammation. It stands to reason that a similar sequel contributes to COVID-19-associated neuropsychiatric complications.

The concept of the MGB axis, summarized by the phrase “mens sana, in corpore Sano,” refers to the codependence of gut and brain health based on the bidirectional communication between gut microbiota, and more precisely, the metabolites produced by gut bacteria (and other microorganisms) and the brain. The MGB axis consists of the components of the central nervous system (CNS), the enteric nervous system (ENS), and the autonomic nervous system, linking the gut microbiota to the brain's cognitive and emotional centers [5, 6]. The concept of the MGB axis has been coined following observations that germ-free mice showed altered behavior [7–9] and altered responses to stress [10, 11]. These seminal observations have not only contributed to a “shift in paradigm in neuroscience” almost a decade ago [12] but also revolutionized our understanding of the role of the gut microbiota in mental health and neuropsychiatric disorders [13]. The significance of the MGB axis in mental health and disease has been supported by the observations of gut microbiota dysbiosis in several neuropsychiatric disorders that include anxiety [14], depression [15–18], cognitive frailty, and dementia [19], and neurosensory abnormalities of taste [20] that are increasingly associated with COVID-19. Notably, chronic systemic inflammation has been associated with behavioral and cognitive dysfunctions due to microglia activation and the recruitment of peripheral lymphocytes into the brain, contributing to inflammation that affects neuronal functions [21]. However, the relationship between gut microbiota, the brain, and neuropsychiatric disorders has not been extensively studied in the context of SARS-CoV-2 infection.

The premise presented here, that SARS-CoV-2 infection results in gut dysbiosis and an altered MGB axis that contributes to the neuropsychiatric ramifications of COVID-19, was examined by comparing the changes in gut microbiota composition in individual neuropsychiatric disorders with changes in gut microbiota in COVID-19. This approach is aimed at identifying the shared “microbial signatures” defined as unique microbial communities related to disease states [22]. Searching for a link between neuropsychiatric disorders and COVID-19 in the gut microbiota composition is supported by the emerging evidence for COVID-19 as a gastrointestinal (GI) infection and several clinical studies documenting gut dysbiosis in COVID-19 patients [23]. The author proposes that the shared microbial signature provides the link between neuropsychiatric disorders and neuropsychiatric ramifications of COVID-19, including symptoms of long-COVID.

Data presented here was compiled from the original and review articles selected from the PubMed base and primarily published during the past two years. Data on COVID-19 were derived from clinical studies due to the lack of relevant small animal models of SARS-CoV-2 because of a low homology between human and rodent viral receptor, angiotensin cleaving enzyme-2 [24]. The existing golden hamster model of COVID-19 has been primarily used in the context of COVID-19 as a respiratory infection. Clinical data on neuropsychiatric disorders are supplemented by animal data comparing symptoms, physiological abnormalities, and current mechanistic theories supporting the premise.

The overreaching goal of this article was to examine and compare specific changes in the gut microbiota, the “microbial signature” in neuropsychiatric disorders, and COVID-19 that may provide the link between COVID-19 and neuropsychiatric disorders. Such a link may allow repurposing the existing therapies to manage short-term neuropsychiatric ramifications of COVID-19 and post-COVID conditions. Therefore, the article concludes by reviewing current probiotic-based therapies in neuropsychiatric disorders and future treatment recommendations for COVID-19-associated neuropsychiatric disorders.

## 2. COVID-19-Associated Neuropsychiatric Disorders, A New Crisis

The rise in global COVID-19 rates has been accompanied by an increase in the prevalence of significant neuropsychiatric disorders. Some reports suggest that more than 80% of hospitalized patients show neurologic symptoms [4]. A more recent report indicates that these complications are not limited to patients with severe COVID-19 [3], and neurological disorders have been observed in asymptomatic COVID-19 individuals [25].

Currently recognized by WHO, COVID-19-associated neuropsychiatric disorders include psychiatric disorders such as generalized anxiety disorder (GAD; [26, 27]), major depressive disorders (MDD; [17, 26]), posttraumatic stress disorder (PTSD; [27]), neurologic disorders [25] such as encephalitis [28], encephalopathies [27, 29], Parkinson's disease (PD), and Guillain-Barré syndrome (GBS; [30]), neurodegenerative disorders such as multiple sclerosis (MS; [31–34]), and neurosensory disorders, loss of smell (anosmia), and loss of taste (dysgeusia; [4]). The association of Alzheimer's disease (AD) and COVID-19 is complicated, although growing evidence of COVID-19-associated cognitive frailty with delirium [35] may be related to a future increase in AD.

Many acute neurological complications, such as encephalitis and encephalopathy, are often associated with psychiatric symptoms [36]. Some individuals experience delirium consisting of confusion, disorientation and agitation, and memory loss [37]. Others experience confusion and prolonged unconsciousness [36] associated with stroke and encephalitis involving brain and spinal cord neurons [27]. Other neurological symptoms include myalgia, headache, acute myelitis, ischemic stroke, intracerebral hemorrhage, acute myelitis, hemiplegia, myositis, and rhabdomyolysis [38]. While hypoxic and ischemic stroke may be precipitated by lung involvement in COVID-19, most neuropsychiatric disorders appear not to be related to respiratory symptoms of infection.

Several factors contribute to the risk of COVID-19-associated neuropsychiatric disorders, including age, gender, ethnicity, diet, and several comorbidities such as hypertension and diabetes. It has been observed that cerebrovascular and neurological complications predominate in older patients [36]. In contrast, acute alterations in mental status [36] and psychiatric disorders such as GAD and depression are more often observed in the younger population [26].

Gender differences in innate and adaptive immune responses [39] are observed in neuropsychiatric disorders. Women demonstrate more robust immunological responses, while men are more likely to develop autoimmune and neurological complications. Some of the differences in immune responses are driven by estrogen levels. However, no gender-related differences were observed in a recent study on COVID-19-associated GAD and depression [26]. Due to the limited number of cases studied, gender differences in risk and disease progression have not been examined. Notably, gender differences in neuropsychiatric disorders may be related to the sexually dimorphic nature of the gut microbiota [40]. Thus, the gender effect should be considered in future studies.

## 3. Shared Gut Microbial Signatures with Deficiency of Short-Chain Fatty Acid-Producing Bacteria: A Link between Neuropsychiatric Disorders and Neuropsychiatric Ramifications of COVID-19?

The concept of “microbial signatures” [22] was used in this review to define a possible link between changes in gut microbiota composition associated with neuropsychiatric disorders and gut microbiota composition in COVID-19. Comparing gut microbial signatures in neuropsychiatric disorders and COVID-19 revealed gut dysbiosis and several similarities: decreased bacterial diversity and richness, depletion of beneficial anti-inflammatory symbiotic bacteria, and specifically short-chain fatty acids- (SCFA-) producing bacteria, and an increase in opportunistic pathogens. Therefore, it is being proposed that the association between COVID-19 and neuropsychiatric disorders may be related to a shared gut microbial signature, specifically a shared deficiency of SCFA producing bacteria and their neuroprotective metabolites.

Several lines of evidence support this supposition. Changes in gut microbiome composition have been observed in several neuropsychiatric diseases [14, 17, 27–29, 41, 42]. Furthermore, SCFA-producing gut bacteria can modulate neuroinflammatory and neurodegenerative processes by producing specific metabolites, acetate, propionate, butyrate, and other less abundant SCFAs.

### 3.1. SCFA Producing Bacteria

SCFAs are produced mainly by Bacteroidetes and Firmicutes; Bacteroidetes produce acetate and propionate, while Firmicutes mostly butyrate. Acetate is produced by several bacterial genera, while a restricted group of specific bacteria produces propionate, butyrate, and lactate. The dominant SCFA-producing genera are *Faecalibacterium prausnitzii* and *Roseburia intestinalis*. *Akkermansia muciniphila*, *Illonella parvula*, *Bacteroides eggerthii*, *Bacteroides fragilis*, *Ruminococcus bromii*, and *Eubacterium dolichum* are responsible for the production of propionate from mucin. The main butyrate-producing bacteria are *Faecalibacterium prausnitzii*, *Clostridium septum*, *Eubacterium rectale*, *Roseburia* spp., *Eubacterium hallii*, *Anaerostipes* spp., and *Ruminococcus bromii* [43, 44]. *Collinsella* spp. is involved in the production of lactate [45].

SCFAs are primarily derived from intestinal microbial fermentation of nondigestible carbohydrates (NDC), also referred to as dietary fibers such as oat and wheat bran, cellulose, pectin, and inulin; 90-95% SCFAs are produced in the colon. Butyrate can also be produced from acetate and bovine milk. Another SCFA, lactate, is produced from a selected group of rapidly fermented NDC. Additionally, acetate, butyrate, and propionate are also produced by fermentation of branched-chain amino acids [46], and butyrate and propionate can also be synthesized from carbohydrates through glycolysis.

### 3.2. SCFA Local Gut Function

SCFAs mainly produced in the colon are absorbed both through passive diffusion or facilitated by binding to the receptors, monocarboxylate transporter 1 (MCT1), which is a primary transporter for butyrate, and sodium-coupled monocarboxylate transporter 1 (SMCT1). SCFAs are involved in local gut functions, such as energy supply for luminal colon cells, GBB integrity, and an anti-inflammatory status. SCFAs bind to the G-protein coupled receptors (GPCRs) and regulate anti-inflammatory signaling cascades. Butyrate promotes epithelial barrier functions by increasing TJs and AMPs, inducing regulatory T cells (Tregs), and controlling inflammation [21]. SCFAs' anti-inflammatory effects include promoting the synthesis of IL-10, a key anti-inflammatory cytokine in the intestinal mucosa, inhibiting histone deacetylases (HDACs), and promoting epithelial cell repair [44]; inhibition of HDACs triggers gene expression [47]. SCFAs are also natural ligands for GPCRs and specifically identified receptors for free fatty acids 2 and 3 (FFAR 2/3) found in many tissues, including the brain [48]. The deficiency of SCFAs results in increased gut permeability and is associated with bacteria's the translocation and cell wall components, triggering an inflammatory cascade [46].

### 3.3. SCFA Endocrine Function

Some SCFAs bind to GPCR transporters, enter the circulation, and exert effects on multiple tissues, including maintaining glucose homeostasis [46] and regulating systemic inflammatory responses. Butyrate plays a role in immune regulation by inhibiting and nuclear factor kappa beta (NF-*κβ*) activation in macrophages and inhibiting HDACs. Propionate and butyrate are involved in regulatory T cell production, inhibition of HDACs, and expression of TNF-alpha and IL-6 [46]. Butyrate also activates Treg and controls inflammation [21]. SCFAs also modulate the peripheral, autonomic, and somatic nervous systems [46]. Importantly, SCFAs are critical metabolites involved in communication with the brain via MGB. Hence, as discussed below, their deficits may significantly impact brain functions and provide a link between SARS-CoV-2 infection and neuropsychiatric disorders.

### 3.4. SCFA Functions in the Brain

SCFAs enter the brain through the blood-brain barrier (BBB), binding to the MCT receptors. SCFAs exert widespread influence on key neurological and behavioral processes in the brain and reinforce BBB integrity [21]. SCFAs influence neuronal functions and contribute to microglial maturation. They modulate neuronal activity directly via receptors expressed on neurons, interact with microglia, and function in brain immunity [49]. SCFAs regulate gene expression by inhibiting histone deacetylases (HDACs), facilitating neuronal outgrowth, cortical network connectivity, and synaptic plasticity through modulation of microglial activation. SCFAs facilitate the expression of anti-inflammatory genes in the immune cells, promoting T lymphocyte differentiation and increasing the response to inflammation [50]. SCFA activity in the brain is exerted by binding to the G protein-coupled receptors (GPCRs) involved in several neurological processes, specifically by the free fatty acid receptor (FFAR) 2 and FFAR3 involved in maintaining energy and immune homeostasis [48]. Acetate modulates the levels of inflammatory cytokines, while propionate and butyrate are involved in the activation of FFAR2 and FFAR3 [21] in the paraventricular nucleus, lateral hypothalamus, and arcuate nucleus. Butyrate inhibits microglial activation and secretion of proinflammatory cytokines and induces morphological and functional changes in microglia. Butyrate inhibits HDACs, promotes histone hyperacetylation in the hippocampus and frontal cortex, enhances learning and memory, and exerts antidepressant effects [51].

SCFAs also modulate the levels of neurotransmitters and neurotrophic factors [21]. Acetate modulates glutamate, glutamine, and GABA levels in the hypothalamus; propionate and butyrate influence intracellular potassium levels. SCFAs regulate the expression of tryptophan 5-hydroxylase 1, involved in the synthesis of serotonin, and tyrosine hydroxylase involved in the biosynthesis of dopamine, noradrenaline, and adrenaline. SCFAs modulate the expression of trophic factors involved in learning and memory, such as brain-derived neurotrophic factor (BDNF), nerve growth factor (NGF), N-methyl-D aspartate receptor subunit 2B, serotonin transporter, and neuropeptide Y system. SCFAs show an effect in several neuronal functions such as sleep and promote memory consolidation [21].

Importantly, SCFAs have been recognized as crucial gut bacterial metabolites involved in signaling along the MGB axis. Hence, their deficit may have a significant impact on the brain.

## 4. Gut Microbial Signature in Neuropsychiatric Disorders

Gut microbiota dysbiosis, referred to as an imbalance of gut microorganisms associated with the disease, results in altered levels of bacterial metabolites communicated to the brain via the MGB axis. Most of the neuropsychiatric pathologies observed in COVID-19 have been independently associated with gut dysbiosis.

Gut dysbiosis has been reported in psychiatric disorders such as anxiety [14], depression [15, 17, 52], neurological disorders such as encephalitis [28, 41, 42], acute encephalopathy [27, 53], acute ischemic stroke [29], neurodegenerative disorders such as AD [54, 55], cognitive frailty and dementia [19], and neurosensory abnormalities such as altered taste [20]. A succinct summary of the data on gut microbial composition derived from the studies of human fecal matter is presented below and in Table 1(a). It is important to point out that most human gut microbiota studies utilize fecal matter that is related yet different from the colonic composition that requires more invasive procedures [53].

### 4.1. Psychiatric Disorders

Analysis of fecal samples from patients with a generalized anxiety disorder (GAD) revealed decreased microbial diversity and richness and a distinct metagenomic composition with reduction of several SCFA-producing bacteria such as *Ruminococcus gnavus*, *Fusobacterium* spp., *Faecalibacterium* spp., *Eubacterium rectale*, *Sutterella* spp., *Lachnospira* spp., and *Butyricicoccus* spp. Reduced synthesis of SCFAs was associated with disruption of the gut-blood barrier (GBB; [51]). On the other hand, there was an overgrowth of bacteria with proinflammatory properties such as *Fusobacterium* spp., *Ruminococcus gnavus*, and *Escherichia-Shigella* [56].

Analysis of fecal samples in patients with major depressive disorder (MDD) showed a significant decrease in *Bifidobacterium* spp. and *Lactobacillus* spp. compared to controls [57]. A more recent study of women with depression showed decreased SCFAs, acetate, and propionate inversely correlated with the severity of depression and a decrease in caproic acid. Additionally, tryptophan and serotonin levels were decreased [17]. These observations suggest a decrease in SCFA-producing bacteria, but information on the bacterial species was not provided.

Altered gut microbiota appears to be involved in depression, as suggested by animal studies [17].

### 4.2. Neurologic Disorders

Prominent gut microbiota dysbiosis is reflected in altered fecal microbiota in patients with autoimmune anti-NMDAR encephalitis [42]. Anti-NMDAR encephalitis, associated with neurological symptoms such as seizures and psychiatric symptoms such as anxiety, memory dysfunction, and irritability [28], is caused by inflammation of the brain parenchyma. Fecal samples showed increased microbial diversity and depletion of commensal genera, such as *Prevotella_6* spp., *Bifidobacterium* spp., *Faecalibacterium* spp., and other SCFA-producing bacteria. On the other hand, there was an increase in *Fusobacterium* spp. considered pathobionts, especially in the older population [42]. Another more extensive study of fecal microbiota showed gut microbiota dysbiosis characterized by decreased alpha diversity index and microbial richness accompanied by intestinal permeability damage [41]. The microbial composition showed a distinct decrease in SCFA-producing bacteria such as *Faecalibacterium* spp., *Roseburia* spp., *Lachnospira* spp., *Ruminococcus* spp., and *Coprococcus* spp. but increased in *Proteobacteria*. There was also an alteration in tryptophan metabolism that may contribute to mucosal dysfunction. Clinical data was supported by observations derived from human to mouse fecal microbiota transplantation studies [41].

Acute hepatic encephalopathy, manifested by changed personality, confusion, disorientation, delirium, psychosis, and thrombotic predisposition [27], has been associated with gut dysbiosis, changes in bacterial metabolites, inflammation, and increased permeability of the GBB [53]. Changes in gut microbiota observed in acute hepatic encephalopathy included decreased SCFA-producing bacteria *Faecalibacterium* spp., *Roseburia* spp., *Enterococcus* spp., and *Bifidobacterium* spp. [53, 58]. Gut microbiota dysbiosis was also observed in other encephalopathies with cerebrovascular symptoms such as ischemic stroke, intracerebral hemorrhage, stroke venous sinus thrombosis, and proinflammatory coagulopathic state. Furthermore, it has been suggested that gut microbiota dysbiosis can be a risk factor for acute ischemic stroke (AISs; [29]). Analysis of fecal samples from an acute ischemic stroke showed a deficit of SCFA-producing bacteria: *Roseburia* spp., *Bacteroides* spp., Lachnospiraceae, *Faecalibacterium* spp., *Blautia obeum*, and *Anaerostipes* spp. that were negatively correlated with stroke severity and prognosis. On the other hand, there was an overgrowth of opportunistic pathogens such as Enterobacteriaceae, Porphyromonadaceae, Lactobacillaceae families, and *Akkermansia* spp. [59].

A meta-analysis of gut microbiota from 223 PD patients and 137 controls from Finland, Russia, USA, Germany, and Japan, showed decreased SCFA-producing bacteria *Roseburia* spp., *Faecalibacterium* spp., and *Lachnospiraceae ND3007*; the results were confirmed by studies in other countries [60]. Furthermore, it has been suggested that a decrease in butyrate synthesizing *Roseburia* spp. and *Faecalibacterium* spp. may contribute to neuroinflammation in PD [60]. On the other hand, there was an increase in *Akkermansia*, associated with intestinal mucin degradation, and *Catabacter*, and *Akkermansiaceae.*

Guillain-Barre Syndrome (GBS), an autoimmune disorder of the peripheral nervous system with gastrointestinal and neurological symptoms, is caused by *Campylobacter jejuni* and leads to gut dysbiosis [66]. Gut dysbiosis and changes in immune responses may be involved in the pathogenesis of GBS that is associated with symptoms such as diarrhea, loss of reflexes, weakness of limbs, and respiratory muscles [67].

### 4.3. Neurodegenerative Disorders

MS, a chronic inflammatory, demyelinating, and degenerative CNS disease, is associated with gut microbiota dysbiosis. Characteristic changes in gut microbiota observed in MS include a decrease in several SCFA-producing bacteria, *Parabacteroides* spp., *Bacteroides stercoris*, *Bacteroides coprocola*, *Bacteroides coprophilous*, *Prevotella copri*, *Haemophilus*, *Sutterella* spp., *Adlercreutzia* spp., *Collinsella* spp., *Coprobacillus* spp., *Lactobacillus* spp., *Clostridium* spp., *Anaerostipes* spp., *Faecalibacterium* spp., and *Clostridium* spp. [55]. Comparison of fecal bacterial composition with and without AD diagnosis showed a decreased microbiota diversity and richness and a profile distinct from age- and sex-matched individuals. A decrease was detected in SCFAs producing *Clostridium*, *Turicibacter* spp., *Bifidobacterium* spp., and *Adlercreutzia* spp. On the other hand, there was an increase in proinflammatory *Bacteroides* spp. and *Alistipes* spp. [61]. Microbiota analysis of fecal samples from patients with cognitive frailty and dementia showed a reduction in microbiota diversity and reduced *Eubacterium rectale* but increased *Escherichia/shigella* [19]. Evidence derived from AD animal models suggests that changes in gut microbiota may be related to cognitive symptoms [19].

### 4.4. Neurosensory Disorders

The risk of anosmia (complete loss of smell) and hyposmia (partial loss of smell) has been linked to the disturbance in the nasal microbiota [68]. However, in addition to the classical olfactory, additional extra nasal receptors in the gut respond to the odorants produced by bacteria and are impacted by gut dysbiosis [69]. Importantly, gut odorants have been shown to control emotions such as fear. Similarly, dysgeusia is associated with abnormal activity of taste receptors (T2Rs) located in the oral cavity where they detect bitter taste; T2Rs are also present in the colon where they function as chemoreceptors in the regulation of GI functions such as GI motility, appetite, nutrient uptake, and fluid secretion. T2Rs, both in the mouth and the colon, are regulated by gut metabolites [20]. T2R dysregulation due to gut microbiota dysbiosis is observed in obesity [20, 70]. Interestingly, T2Rs interact with SCFAs considered bitter bacterial metabolites, and the deficiency in SCFAs could increase dysgeusia [20].

### 4.5. Stress

Stress is an overreaching insult to our organism, several underlying pathologies; it is pervasive in situations such as COVID-19 pandemics. It independently contributes to gut dysbiosis and altered activity of the MGB axis. The effect of prolonged stress combining physical exertion, psychological stress, sleep deprivation, and environmental stressors during military combat showed changes in gut microbiota and increased intestinal permeability. Analysis of fecal samples showed increased alpha diversity but not bacterial richness and a decrease in the relative abundance of anti-inflammatory *Bacteroides* spp. and SCFAs producing *Faecalibacterium* spp., *Collinsella* spp., and *Roseburia* spp., but an increase in proinflammatory *Sutterella* spp., and the relative abundance of deleterious *Peptostreptococcus* spp., *Staphylococcus* spp., *Peptoniphilus* spp., *Acidaminococcus* spp., and *Fusobacterium* spp. [62].

For summary, a review of the gut microbiota composition in individual neuropsychiatric disorders identified shared changes in microbial communities, which we will refer to as the “microbial signature” of neuropsychiatric disorders. The “microbial signature” of neuropsychiatric disorders is defined by a decreased microbial diversity and richness, decreased health-promoting anti-inflammatory commensal bacteria with a specific decrease in the abundance of SCFA producing bacteria, and an increase in opportunistic proinflammatory pathogens. Importantly, SCFA producing bacteria results in a deficiency of critical bioactive microbial metabolites with anti-inflammatory functions recognized as critical for MGB axis signaling. Independently, the deficiency of SCFAs is associated with brain inflammation and neuropsychiatric disorders.

It is important to acknowledge that defining the “microbial signature” based on limited data may be an oversimplification due to high within– and between-subject variability due to comorbidities, age, gender, ethnicity, diet, population density, and limitations of the current methodologies [71]. Very few existing studies consider different factors that may impact gut microbiota composition. Nevertheless, the concept of gut microbial signature facilitates the comparison of gut dysbiosis in neuropsychiatric disorders and SARS-CoV-2 infection, while future studies are needed to validate this approach.

## 5. Gut Microbial Signature in COVID-19

Relatively few studies have examined the changes in the gut microbiota associated with COVID-19. However, limited evidence suggests a potential role of gut microbiota in the susceptibility, progression, and severity of COVID-19 [63, 64]. Indeed, GI symptoms observed in COVID-19 are closely related to gut dysbiosis [72]. Several groups have used human fecal samples to examine changes in gut microbiota in COVID-19. These studies show a decrease in biodiversity and the number of health-promoting commensal bacteria such as *Lactobacillus* spp. and increased the proinflammatory bacteria such as Clostridiales family [73], a pattern resembling microbiota changes associated with inflammatory bowel disease (IBD). Changes in the gut microbiota have been shown to persist after clearance of SARS-CoV-2 and resolution of respiratory symptoms [65]. A summary of specific changes in gut microbiota composition in COVID-19 patients relative to control subjects is presented below and in Table 1(b).

A study involving a small group of hospitalized patients in China showed a significant reduction in bacterial diversity and richness, accompanied by a dramatic reduction in several SCFA-producing bacteria, including *Agathobacter* spp., *Fusicatenibacter* spp., *Roseburia* spp., and *Ruminococcaceae UGC-013* in COVID-19 patients compared to healthy controls. On the other hand, there was an increase in opportunistic pathogens *Streptococcus* spp., *Rothia* spp., *Veillonella* spp., *Erysipelatoclostridium* spp., and *Actinomyces* spp. Furthermore, the levels of *Fusicatenibacter* spp., *Romboutsia* spp., *Intestinibacter* spp., *Actinomyces* spp., and *Erysipelatoclostridium ramosum* distinguished between COVID-19 and healthy controls and several bacterial species were associated with fecal viral load [74]. Examination of serial stool samples from hospitalized patients with COVID-19 and non-COVID-19 individuals recruited in Hong Kong and corrected for the use of antibiotics showed COVID-19-associated decrease in SCFA producing bacteria with known immunomodulatory properties such as *Faecalibacterium prausnitzii*, *Eubacterium rectale*, *Bifidobacterium* pseudocatenulatum, *Ruminococcus bromii*, *Blautia obeum*, and *Dorea formicigenerans*, during hospitalization and four weeks of recovery. On the other hand, there was enrichment in *Ruminococcus* sp. *5_1_39BFAA*, *Prevotella copri*, *Dorea longicatena*, *Streptococcus salivarius*, and *Eubacterium hallii* concordant with disease severity. Furthermore, the gut microbiota composition of discharged COVID-19 patients differed from that of the general population, showing lower levels of symbiotic bacteria and gut dysbiosis even after the disappearance of respiratory symptoms and clearance of SARS-CoV-2 in the throat swabs [64]. Another study involving a small number of COVID-19 antibiotics-naïve patients showed decreased SCFA producing bacteria, including *Faecalibacterium prausnitzii*, *Bacteroides dorei*, *Bacteroides thetaiotaomicron*, *Bacteroides massiliensis*, and *Bacteroides ovatus*. A decrease was also observed in *Alistipes onderdonkii* involved in the metabolism of tryptophan to serotonin [65]. On the other hand, there was an enrichment in opportunistic fecal pathogens including *Coprobacillus*, *Clostridium ramosum*, *Clostridium hathewayi*, *Actinomyces viscosus*, and *Bacteroides nordic* compared to controls. Interestingly, four species of *Bacteroides phylum*, including *Bacteroides dorei*, *Bacteroides thetaiotaomicron*, *Bacteroides missiles*, and *Bacteroides ovatus*, associated with downregulation of ACE2 expression, showed an inverse correlation with increased SARS-CoV-2 load [65]. Furthermore, high *Clostridium ramosum*, *Clostridium hathewayi*, and *Coprobacillus levels* were consistently associated with COVID-19 severity. The gut microbiota changes persisted during hospitalization and continued even after clearance of SARS-CoV-2 in nasal swabs and resolution of respiratory symptoms [65].

Changes in gut microbiota in hospitalized COVID-19 patients also affected the fungal mycobiota. COVID-19 fungal mycobiome was characterized by reduced fungal diversity and richness and increased fecal fungal pathogens compared with controls [65].

For summary, although there is limited data on gut microbiota composition in COVID-19, an emerging COVID-19 “microbial signature” can be characterized by decreased bacterial diversity and richness, by a decreased beneficial symbiont, with a specific decrease in the abundance of SCFA-producing bacteria, and increased opportunistic pathogens.

## 6. Gastrointestinal Aspects of SARS-CoV-2 Infection

COVID-19 is increasingly recognized as a GI infection [23]. Supporting evidence points to GI symptoms such as diarrhea, nausea, and vomiting [75–77], affecting over half of the COVID-19 patients [78]. Notably, a small number of COVID-19 patients show only GI symptoms such as diarrhea, anorexia, nausea, and vomiting [76, 79]. The oral route of SARS-CoV-2 infection is supported by endoscopy showing viral particles in the GI [76, 79, 80] and significant epithelial damage [81]. The presence of SARS-CoV-2 RNA [82], viral particles, and live viruses was observed in the fecal samples of COVID-19 affected individuals [74, 80, 83]. Importantly, high levels of infectious virus are present in the intestinal lumen of infected asymptomatic patients [84].

Gastrointestinal aspects of COVID-19 infection are further supported by a high level of expression of SARS-CoV-2 receptor, ACE-2, in the GI tract [85], including the esophagus, duodenum, ileum [76, 79, 86], and colon [76, 79]. ACE-2 receptors are more abundant in the GI tract than in the respiratory tract [85] and highly expressed in the colon and regulated by the microbiota [14].

ACE-2 receptors are components of the renin-angiotensin-aldosterone system (RAAS), regulating blood pressure and fluid dynamics. In addition to the systemic functions and local RAAS-dependent activity, ACE-2 is involved in local organ-specific RAAS-independent activity reviewed recently [23]. In the gut, ACE-2 RAAS-independent functions are associated with the bradykinin receptor B1 (BKB1R) axis and with the Mas oncogene (Mas) receptor Ang-(1-7)/Mas axis involved in the acute inflammatory response and sodium-dependent neutral amino acid transporter (B^0^AT1; [87]). B^0^AT1 shares a location with ACE-2 in the small intestine brush border and acts as a chaperone for membrane trafficking of neutral amino acids, mediates the uptake of glutamine and tryptophan, and regulates glucose, fluid, and electrolyte absorption and secretion, and motility. It also promotes anti-inflammatory status, preserves tight junctions (TJs), decreases mucosal cell autophagy, and increases antimicrobial peptides (AMPs) through the mTOR pathway.

ACE-2 receptor also regulates the expression of B^0^AT1 [88].

### 6.1. ACE-2 Deficiency Contributes to the Gut Microbiota Dysbiosis

**Figure 1:**
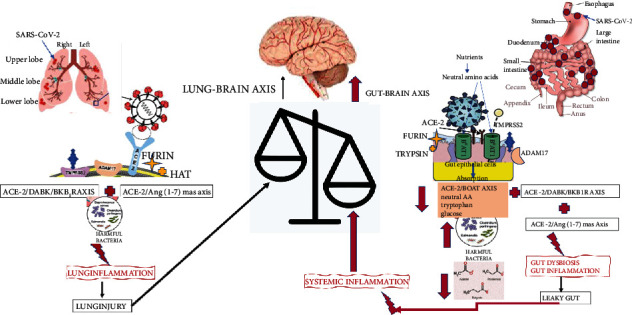
Brain impact of lung vs. gut ACE-2 deficiency. Lung ACE-2 deficiency increases local inflammation by upregulating ACE-2/DABK/BKB1R and downregulating ACE-2/Ang2/Ang-(1-7)/Mas axis; increased lung inflammation results in lung injury and exerts a hypoxic effect on the brain. Gut ACE-2 deficiency upregulates ACE-2/DABK/BKB1R and downregulates ACE-2/Ang2/Ang-(1-7)/Mas axis but also downregulates the formation of ACE-2/B^0^AT1 complexes and intestinal uptake of neutral amino acids such as glutamine and tryptophan, critical to T-cell functions. ACE-2 deficiency in the gut contributes to gut dysbiosis, inflammation, increased permeability of the gut-blood barrier, and systemic inflammation that impacts the brain.

Upon binding to ACE-2, SARS-CoV-2 sequesters the receptor and downregulates the level of luminal ACE-2, resulting in the deficiency of ACE-2. Gut ACE-2 deficiency impacts several processes, illustrated in Figure 1, such as nutrient transport, microbial regulation, local immunity, and gut-brain barrier (GBB) permeability [89]. Increased GBB permeability enhances systemic inflammation and may facilitate cytokine storms [90].

ACE-2 deficiency results in downregulation of ACE-2/B^0^AT1 complexes and decreased intestinal uptake of neutral amino acids such as glutamine and tryptophan required for serotonin synthesis [90]. These amino acids also activate toll-like receptors (TLRs) signaling and NF-*κβ*. They are critical to T cell function and innate and adaptive immunity [90]. The deficit of ACE-2 decreases activation of the mTOR pathway, reducing the secretion of AMPs, alters the gut microbiota, and increases susceptibility to inflammation [49]. Impaired amino acid transport and reduced secretion of AMPs affect innate immunity and contribute to colitis or IBD-like symptoms [88]; reduced levels of AMPs lead to dysbiosis in COVID-19 [14]. Accumulation of neutral amino acids in the intestinal lumen brings about microbiota changes, immune dysregulation, and Hartnap disease-like symptoms [88]. A deficit in tryptophan affects incretins involved in maintaining glucose homeostasis and contributes to hyperglycemia.

While the review focuses on the gastrointestinal nature of SARS-CoV-2 infection and the link between gut dysbiosis and the altered MGB axis, one cannot dismiss the respiratory nature of the virus, its effect on lung injury, and its impact on the lung-brain axis. It has been suggested that lung injury contributes to brain hypoxia and CNS injury in COVID-19. Based on the early and limited neuroimaging data, hypoxic brain damage may result in hypoxic-ischemic encephalopathy, demyelination, oligodendroglia cell injury, and BBB dysfunction [91]. Furthermore, the nasal route of infection with SARS-CoV-2 may lead to the dysbiosis of lung-specific microbiota and contribute to lung inflammation observed in COVID-19 patients. Interestingly, several chronic lung disorders, respiratory infections, and respiratory diseases such as asthma and cystic fibrosis have been associated with gut microbiota and lung microbiota dysbiosis [92]. Gut microbiota may play a critical role in regulating immune responses and lung microbiota via the gut-lung axis. Immune regulation may involve systemic dissemination of metabolites such as SCFAs produced in the colon that reach the lungs via the bloodstream and exert anti-inflammatory properties [92]. Changes in lung microbiota are observed in obstructive pulmonary disease (COPD) and include lower bacterial diversity, decreased health-promoting commensal bacteria, Firmicutes, and increased disease-associated Proteobacteria [93]. Importantly, changes in the lung microbiota identified in COVID-19 patients showed enrichment with bacteria found typically in the intestinal tract [94], supporting the notion that COVID-19-associated gut microbiota dysbiosis contributes to changes in lung microbiota dysbiosis.

## 7. Altered MGB Axis Signaling in Neuropsychiatric Disorders and COVID-19

The concept of the MGB axis, defined as the bidirectional communication between the gut microbiota and brain, has been verified in both animal and numerous preclinical and clinical studies, underscoring the involvement of MGB in maintaining health and contributing to various neuropsychiatric disorders. The disruption of GMB activity due to gut dysbiosis has been linked to several psychiatric disorders such as anxiety and depression [95, 96], neurological disorders such as PD, and neurodegenerative disorders such as AD [54, 97]. Disruption of the MGB axis may have a critical impact on the long-term neuropsychiatric ramifications of COVID-19. A review of data on gut microbiota composition in neuropsychiatric disorders and COVID-19 revealed a shared gut microbial signature defined by less diverse and less numerous bacterial communities with a specific decrease in SCFAs and serotonin-producing bacteria.

### 7.1. SARS-CoV-2 Infection Results in Gut Microbiota Dysbiosis due to the Dysregulation of Nutrient Transport

As discussed in previous sections, ACE-2 impacts the gut microbiota by regulating the transport of nutrients such as neutral amino acids and glucose and directly controlling AMP secretion [14]. ACE-2 deficiency in COVID-19 disrupts B^0^AT1-dependent transport of amino acids and serotonin synthesis. The resulting in nutrient deficit leads to gut dysbiosis characterized by a reduction in bacterial diversity combined with decreased beneficial symbionts and increased opportunistic pathogens in COVID-19 patients. Gut microbiota dysbiosis in COVID-19 contributes to increased GBB permeability—the “leaky gut” activates the gut-associated lymphoid system (GALT) and increases inflammation. Gut microbiota dysbiosis is likely responsible for the cytokine storms leading to multiorgan failure. Furthermore, gut microbiota dysbiosis impacts the lung microbiota by enrichment with bacteria found in the intestinal tract [94].

### 7.2. MGB Axis and SCFA Deficiency

**Figure 2:**
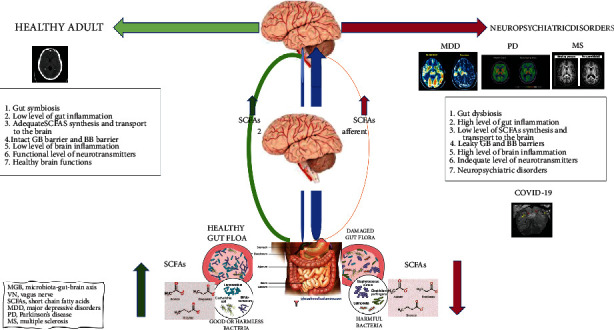
Gut dysbiosis, SCFA deficiency, and altered MGB axis in neuropsychiatric disorders and COVID-19. A schematic representation of a possible link between microbial signature defined by a decreased microbial diversity and richness and a deficiency in SCFA-producing bacteria and neuropsychiatric disorders and COVID-19. MDD: major depressive disorders; PD: Parkinson's disease; MS: multiple sclerosis; MGB: microbiota-gut-brain axis; VN: vagal nerve; SCFAs: short-chain fatty acids; GBB: gut-blood barrier; BBB: brain-blood barrier.

Gut dysbiosis, including deficiency of SCFA and serotonin, is communicated to the brain by the MGB axis through the CNS, the ENS, ANS, and the hypothalamic-pituitary-adrenal (HPA) axis. In both the sympathetic and parasympathetic arms of ANS send afferent signals to the brain via vagal and spinal pathways and efferent signals from the brain to the gut via the HPA axis [5]. The VN is the principal component of the parasympathetic nervous system. VN afferents sense luminal signals by diffusion of bacterial compounds or metabolites such as serotonin and gut hormones [98]. The schematic representation of the altered MGB axis in neuropsychiatric disorders and COVID-19 is presented in Figure 2.

SCFAs are involved in many brain processes, and their deficit results in several critical changes in crucial neurological and behavioral processes that contribute to neuropsychiatric disorders [21]. A deficit in SCFAs contributes to chronic brain inflammation associated with behavioral and cognitive dysfunctions and many brain pathologies. Chronic inflammation involves microglia activation and the recruitment of peripheral lymphocytes into the brain and affects neuronal functions. Increased gut permeability of gut-blood barrier (GBB) due to SCFA deficiency contributes to the translocation of bacterial products, increases cytokine levels, and impacts the BBB integrity. Brain inflammation via cytokine storm is one of the mechanisms of major depressive disorder (MDD). Decreased acetate, propionate, and caproate have been associated with depression. Lower acetate levels reduce butyrate involved in hippocampal microglia activation and contribute to neuroinflammation and depression [17, 18]. SCFA's deficiency may contribute to degenerative processes as SCFAs modulate neuronal plasticity by promoting cortex connectivity and moderating brain-invading lymphocytes' effects on microglial functions [50].

SCFAs function as cognitive enhancers by inhibiting the activity of HDACs and promoting hyperacetylation of histones in the hippocampus, frontal cortex. Deficit of SCFAs in the brain may affect psychological functioning and contribute to depression [98]. The deficit in brain serotonin levels further contributes to a decline in memory and mood and contributes to the pathophysiology of neuropsychiatric disorders.

Furthermore, SCFA deficiency decreases the expression of trophic factors involved in learning and memory, such as BDNF, NGF, and neuropeptide Y system. SCFA deficiency affects several neuronal functions, such as sleep and memory consolidation [21]. SCFAs' deficiency results in decreased levels of glucagon-like peptide (GLP1), promoting memory and learning, and peptide yy (PYY) with antidepressive properties. SCFA deficiency thus results in increased depressive-like behavior [51]. SCFA deficiency has been implicated in many neuropsychiatric disorders associated with COVID-19, such as depression, bipolar disorder, and cognitive functions in animals and humans [51].

## 8. Future Therapies for COVID-19-Associated Neuropsychiatric Disorders: Supplementation with Probiotics, Prebiotics, and Synbiotics to Improve Cognitive Functions

SARS-CoV-2 infection results in gut microbiota dysbiosis due to dysregulated transport of intestinal nutrients. Gut microbiota dysbiosis increases gut-blood barrier permeability, activates GALT, and increases inflammation. Gut microbiota dysbiosis is likely responsible for cytokine storm and systemic inflammation leading to multiorgan failure, including the lungs and the brain. Altered gut microbiota impacts lung microbiota in COVID-19 with a change in composition involving an increased proportion of gut species [6]. Altered gut microbiota results in the altered composition of the metabolites, such as SCFA deficiency, crucial for brain health. Clinical and animal data support the notion of SCFA deficiency as a critical contributor to COVID-19-associated neuropsychiatric disorders. This perspective of SCFA deficiency and disruption of the MGB axis by SARS-CoV-2 may facilitate repurposing the existing therapies, such as SCFA or probiotic-based supplementation, to manage the neuropsychiatric ramifications of COVID-19.

### 8.1. SCFA Supplementation

In 2017, the systemic availability and metabolism of colonic-derived SCFAs were quantified in healthy individuals [51]. The existing clinical and animal data suggest that SCFA supplementation may be therapeutic in a range of neurologic and neuropsychiatric conditions. The effect of SCFAs on psychosocial stress and fear task response in healthy men showed that colonic administration of physiological doses of SCFA results in increased plasma SCFA levels and significantly attenuates the cortisol response to acute psychosocial stress [99].

In animals, SCFAs can be delivered orally to reduce systemic inflammation [100]. In humans, SCFAs can be administered orally as tablets releasing SCFAs into the colon or administered by enemas resulting in decreased systemic inflammation. Delivery of acetate directly to the distal colon by rectal infusion in female subjects resulted in a significant increase in plasma tumor necrosis factor- (TNF-*α*-) dependent immune cell signaling and a decrease in systemic inflammation. Since acetate is the primary SCFA in circulation, it has the most potent effect on inflammation [101].

Significantly, FTM from patients with autoimmune encephalitis to microbiota-depleted mice resulted in behavioral and cognitive impairments [65]. SCFA pretreatment significantly attenuated neurologic, immune, and behavioral changes observed in sepsis-associated encephalopathy in mice [102]. Other animal studies suggest that SCFAs reduce inflammation, axonal damage [50], and the severity of experimental autoimmune encephalomyelitis in MS models by suppressing demyelination and enhancing remyelination [21]. Butyrate supplements were shown to induce antidepressant and anxiolytic behaviors in mouse models of depression, and the effect was associated with reduced plasma corticosterone levels [73]. Butyrate supplementation also improved cognition and memory functions in rodent AD models [21]. SCFAs proved to be effective in recovering memory functions in associative learning.

### 8.2. Probiotic Supplementation

Maintaining gut homeostasis and the use of probiotics as a treatment option in patients with severe COVID-19 has been recommended by China's National Health Commission (CNHC; 5th edition; [74, 99]). In February 2020, the CNHC suggested using probiotics in patients with severe COVID-19. The prebiotics or probiotics were suggested in COVID-19 infected patients to reinforce colonic microbiota in antibiotic-treated patients with diarrhea [103] and reduce the risk of secondary infection [102]. Two randomized controlled trials showed that probiotic treatment (*Lactobacillus GG*, *Bacillus subtillis*, and *Enterococcus faecal*is) decreased ventilator-associated pneumonia. However, the data on probiotic-based therapies has been limited and has provided mixed results [104].

On the other hand, probiotic-based treatments have been employed in several COVID-19 comorbidities, like obesity and diabetes. A closer examination of the probiotics used in several studies and clinical trials reveals that they are composed of SCFA-producing bacteria. Human-origin probiotic cocktail containing *Lactobacillus* and *Enterococcus* strains isolated from healthy infants and delivered by oral gavage to mice increased SCFA synthesis, including butyrate, and inhibited the growth of uropathogenic strains of *Enterobacteriaceae.* Inoculation of the same cocktail in human feces also increased SCFA production. The above observations support a potential therapeutic role of these probiotics in diseases with SCFA deficiency [105], including COVID-19.

To screen the potential microbial strains with probiotic attributes, AAO and the World Health Organization (WHO) have established criteria such as tolerance to oro-gastrointestinal transiting, including high acid and bile concentration, and adherence to the human gastrointestinal mucosa. Bacterial strains must be of human origin and susceptible to common antibiotics without virulence factors and antibiotic resistance; they should be isolated from the local population. Notably, the used probiotics are composed of lactic acid bacteria (LAB) and Bifidobacteria spp. Both Lactobacillus and Enterococcus strains are found naturally in the human intestine.

Several human trials tested the effects of probiotics on SCFA production by human intestinal microbiota [106] in healthy controls, obesity, and gastrointestinal disorders. A four-week treatment of healthy adults with *Bifidobacterium lactis LAFTIB94* resulted in increased *Bifidobacterium* spp. but did not affect SCFA concentration. A six-month treatment of obese children with *Lactobacillus casei* increased *Bifidobacterium spp.* and acetic acid concentration in the feces. In contrast, a 10-day treatment with *Lactobacillus rhamnosus GG* of children with bacterial infections resulted in increased propionic acid.

### 8.3. Prebiotics

The growth and the metabolic activity of probiotic microorganisms can be enhanced by nondigestible carbohydrates (NDCs), also referred to as dietary fibers. NDCs include resistant starch (RS) and plant-derived NDCs. The plant-derived NDCs include nonstarch polysaccharides (NSP), oligosaccharides, disaccharides, and monosaccharides [106]. A study that explored the efficacy of different prebiotics compared butyrate production by supplementing the diets of healthy young adults for two weeks with resistant starch from potatoes (RPS), resistant starch from maize (RMS), inulin from chicory root, and rapidly digestible corn starch. This comparison showed that RPS resulted in the most increase in SCFAs, including butyrate, and an increase in SCFA producing bacteria, *Ruminococcus bromii*, and *Clostridium chartatabidum* [107].

### 8.4. Synbiotics

The combination of probiotics with prebiotics called synbiotics has been shown to increase the level of predominant bacteria and the production of SCFA using a four-stage model of the human colon [108]. Another study examined the efficacy of NSP derived from oats in combination with a probiotic Bacteroides licheniformis, facilitated SCFA (lactic and succinic acids) synthesis by other bacteria [109].

### 8.5. Candidate Probiotics for COVID-19 Supporting Cognitive Functions

The use of probiotics to enhance cognitive functions is supported by animal studies that identified several probiotic candidates, including *Bifidobacterium longum*, *Bifidobacterium breve*, *Bifidobacterium infantis*, *Lactobacillus helveticus*, and *Lactobacillus rhamnosus*. *Lactobacillus plantarum* and *Lactobacillus casei* improve CNS functions such as anxiety, depression, affective, stress, and memory [110]. Chronic treatment with a butyrate-producing bacterium, Bifidobacterium longum, has been shown to ameliorate anxiety-like behavior in mice [111]. Significantly, the strains that support cognitive and behavioral function identified above and used with doses between 109 and 1010 colony-forming units for two weeks in animals and four weeks in humans attenuated psychiatric disorders such as anxiety, depression, and memory deficit. Furthermore, SCFA-producing strains, Bifidobacterium longum, Bifidobacterium breve, Bifidobacterium infantis, Lactobacillus helveticus, Lactobacillus rhamnosus, Lactobacillus plantarum and Lactobacillus casei appear to be most effective in improving CNS functions [112].

The use of probiotics supporting SCFA synthesis opens a gate for future use of other probiotics to restore gut microbiota and the MGB axis. An essential aspect of future probiotic-based therapies in COVID-19 requires careful consideration of various factors that impact human microbiota, such as race, age, diet, and medication. Furthermore, gender is an essential factor affecting the gut microbiota [40], as suggested by animal data showing different effects of FTM on microbiota compositions in adult male and female mice. Human studies support gender dependence between diet and gut microbiota; dietary supplementation with NSPs may result in higher fecal SCFA concentration in males than for females [113]. Notably, SCFAs are decreased in PD in a gender-dependent manner [114]. Thus, race-, age-, diet-, medication-, and gender-dependent microbiota differences must be considered while designing future probiotic-based therapies in COVID-19.

More research is needed to substantiate further the linkages and perspective described in this review and evaluate the potential significance of gut microbiota profiling as a diagnostic tool. Meanwhile, it is prudent to propose changes in dietary recommendations in favor of a high fiber diet or supplementation with SCFAs or probiotics to prevent or alleviate neuropsychiatric complications associated with COVID-19.

## 9. Conclusions

The data presented in this review supports the premise that SARS-CoV-2 infection results in gut dysbiosis and an altered MGB axis that contributes to the neuropsychiatric ramifications of COVID-19. Furthermore, the evidence points to a specific gut microbial signature in COVID-19 defined by decreased diversity and richness of the microbiota and a specific deficit in SCFA-producing bacteria. A similar gut microbial signature is associated with neuropsychiatric disorders. Shared gut microbial signatures with deficiency of SCFA-producing bacteria provide a link between neuropsychiatric and neuropsychiatric ramifications of COVID-19. Future probiotic-based therapies targeting SCFA deficit may prevent or ameliorate neuropsychiatric sequel of SARS-COV-2 infection.

## Figures and Tables

**Table 1a:** (a) Deficiency of SCFA-producing bacteria in neuropsychiatric disorders

Disorder	Decreased SCFA-producing bacteria	Reference	Methodology
Generalized anxiety disorder (GAD)	*Butyricicoccus* spp. *Eubacterium rectale* *Faecalibacterium* spp. *Lachnospira* spp. *Ruminococcus gnavus* *Fusobacterium* spp. *Sutterella* spp.	Jiang et al. [56]	16S rRNA gene pyrosequencing
Major depressive disorder (MDD)	*Bifidobacterium* spp. *Lactobacillus* spp.	Aizawa et al. [57]	16S rRNA sequencing
Anti-NMDAR encephalitis	*Bifidobacterium* spp. *Faecalibacterium* spp. *Prevotella_6* spp. *Coprococcus* spp. *Faecalibacterium* spp. *Lachnospira* spp. *Roseburia* spp. *Ruminococcus* spp.	Gong et al. [42] Chen et al. [41]	16S rRNA gene pyrosequencing 16S rRNA gene pyrosequencing
Acute hepatic encephalopathy	*Bifidobacterium* spp. *Burkholderia* spp. *Enterococcus* spp. *Faecalibacterium* spp. *Roseburia* spp.	Bajaj [58] Rai et al. [53]	Review: 16S rRNA gene pyrosequencing 16S rRNA gene pyrosequencing
Acute ischemic stroke	*Anaerostipes* spp. *Bacteroides* spp. *Blautia obeum* *Faecalibacterium* spp. *Roseburia* spp.	Tan et al. [59]	16S rRNA sequencing
Parkinson's disease (PD)	Faecalibacterium spp. *Lachnospiraceae ND3007 Roseburia* spp.	Nishiwaki et al. [60]	16S rRNA sequencing, followed by meta-analysis
Multiple sclerosis (MS)	*Adlercreutzia* spp. *Anaerostipes* spp. *Bacteroides coprocola* *Bacteroides coprophilous* *Bacteroides stercoris* *Collinsella* spp. *Faecalibacterium* spp. *Lactobacillus* spp. *Parabacteroides* spp. *Provotella copri* *Sutterella* spp.	Schepici et al. [55]	Review: 16S rRNA sequencing
Alzheimer's disease (AD)	*Adlercreutzia* spp. *Bifidobacterium* spp*. Turicibacter* spp.	Vogt et al. [61]	16S rRNA sequencing
Cognitive frailty and dementia	*Eubacterium rectale*	Ticinesi et al. [19]	Review: 16S rRNA sequencing
Stress	*Bacteroides* spp. *Collinsella* spp. *Faecalibacterium* spp. *Roseburia* spp.	Karl et al. [62]	16S rRNA sequencing

**Table 1b:** (b) Deficiency of SCFA-producing bacteria in COVID-19

Decreased SCFA-producing bacteria	Reference	Methodology
*Agathobacter* spp. *Fusicatenibacter* spp. *Intestinibacter* spp. *Romboutsia* spp. *Roseburia* spp. *Ruminococcaceae UGC-013*	Segal et al. [63]	Review: 16S rRNA sequencing, shotgun metagenomic sequencing deep shotgun metagenomic sequencing
*Bifidobacterium* spp. *Blautia obeum* *Dorea formicigenerans* *Eubacterium rectale* *Faecalibacterium prausnitzii* *Bifidobacterium pseudocatenulatum* *Ruminococcus bromii*	Yeoh et al. [64]	Shotgun sequencing of total DNA
*Bacteroides dorei* *Bacteroides thetaiotaomicron* *Bacteroides massiliensis* *Bacteroides ovatus* *Faecalibacterium prausnitzii*	Zuo et al. [65]	DNA flex sequencing
